# Improvement of Therapeutic Efficacy of Oral Immunotherapy in Combination with Regulatory T Cell-Inducer Kakkonto in a Murine Food Allergy Model

**DOI:** 10.1371/journal.pone.0170577

**Published:** 2017-01-20

**Authors:** Yuka Nagata, Takeshi Yamamoto, Michie Hayashi, Shusaku Hayashi, Makoto Kadowaki

**Affiliations:** Division of Gastrointestinal Pathophysiology, Institute of Natural Medicine, University of Toyama, Toyama, Japan; Wayne State University, UNITED STATES

## Abstract

Oral immunotherapy (OIT) has been considered a promising approach for food allergies (FAs). However, the current OIT strategy is limited in terms of the long-term efficacy and safety. We have previously demonstrated that kakkonto, a traditional Japanese herbal medicine, suppresses the occurrence of allergic symptoms in a murine model of ovalbumin (OVA)-induced FA, which is attributed to the induction of the Foxp3^+^ CD4^+^ regulatory T cells. In this study, we established an OIT model using the FA mice with already established allergic symptoms and determined whether kakkonto could improve the efficacy of OIT. The OIT method consisted of initially administrating a very small amount of OVA and slowly increasing the amount. Allergic symptoms decreased in the OIT-treated FA mice. OIT significantly downregulated Th2 immune response-related gene expression in the FA mouse colon, and decreased the level of mouse mast cell protease-1, a marker of mast cell degranulation in the FA mouse plasma. Moreover, the concomitant use of kakkonto significantly enhanced the effectiveness of OIT on the allergic symptoms, and the combination therapy further suppressed the Th2 immune responses and the mast cell degranulation. In addition, OIT significantly increased the population of Foxp3^+^ CD4^+^ regulatory T cells in the FA mouse colon, and this population was further increased by OIT in combination with kakkonto. Furthermore, the combined therapy with kakkonto reduced the expression of RA-degrading enzyme CYP26B1 mRNA in the FA mouse colon. These findings indicated that the combination of OIT with kakkonto represents a promising approach for FA treatment.

## Introduction

Food allergies (FAs) represent an increasingly prevalent human health problem that affects a large proportion of the general population in developed countries [1]. Up to 8% of children and 5% of adults self-reported an allergy to at least 1 food [1, 2]. Despite the increasing prevalence of FA, therapeutic options remain limited [3, 4]. No treatments have been proven to accelerate the development of oral tolerance or to provide effective protection from accidental exposure. The current standard management relies on antigen avoidance and emergency preparedness [4, 5].

Allergen-specific oral immunotherapy (OIT) has been considered a promising potential therapeutic approach for FAs to induce permanent immunological tolerance to food allergens [5–7]. There have been reports of success in several clinical trials of OIT for milk [8, 9], egg [10, 11], and peanut [12, 13] (ClinicalTrials. gov Identifiers in these clinical trials described in S2 Table). However, to date, the available evidence for the effectiveness, risk-benefit ratio and potential long-term consequences of OIT is insufficient to support its use in clinical practice. In addition, the optimal dose and length of therapy is also unclear. Previous studies on OIT have used a variety of doses, and the strategies were heterogeneous, making comparisons among them difficult [5, 14–16]. Moreover, the underlying molecular and cellular mechanisms of OIT remain unclear [5]. To understand the precise mechanisms of OIT and determine whether OIT is effective and safe treatment against FA, an appropriate animal model needs to be established. Until now, however, appropriate animal models for OIT for egg allergies have not been available.

Kakkonto, a traditional Japanese herbal medicine, is commonly used in Japan. The most important aspect contributing to the frequent use of kakkonto is that kakkonto is a highly effective and safe medicine for the treatment of the common cold [17, 18], influenza [19], allergic rhinitis [20] and diarrhea either as the sole source of therapy or in combination with modern Western medicines. We have previously demonstrated that kakkonto suppresses the occurrence of allergic symptoms in a murine FA model [21] and kakkonto induces Foxp3^+^ CD4^+^ regulatory T cells (Tregs) in the colon as a novel mechanism underlying the therapeutic action [22]. It is reported that allergen-specific immunotherapy increases the production of local and systemic Foxp3^+^ CD4^+^ Tregs as an essential step in patients [23, 24] and experimental models [25–28]. Therefore, we hypothesized that kakkonto might have a potential as a therapeutic drug for the treatment of immune diseases induced by the disruption of intestinal mucosal tolerance, such as FAs. In this study, we demonstrated that concomitant use of kakkonto with OIT (OIT+kakkonto) can result in better therapeutic efficacy for OIT using an OIT mouse model established in this study.

## Materials and Methods

### Animals

Four-week-old male BALB/c mice were purchased from Japan SLC, Inc. (Shizuoka, Japan) and RAG-2^-/-^ DO11.10 mice were purchased from Taconic Laboratories (Germantown, NY, USA). All mice were housed in the experimental animal facility at the University of Toyama. This study was approved by the Animal Experiment Committee at the University of Toyama (Authorization No. A2012inm-4 and A2015inm-3) in accordance with the Guide for the Care and Use of Laboratory Animals at the University of Toyama, which is accredited by the Ministry of Education, Culture, Sports, Science and Technology, Japan. All mice were kept under SPF conditions and observed approximately every day for adequacy of food, water, bedding and general overall health conditions especially by monitoring a condition of stool. Consistent with the approvals stipulated by the protocol, all efforts were made to minimize suffering or discomfort to the animals.

### Kakkonto

Kakkonto, a traditional Japanese herbal medicine (Kampo medicine), is approved as an ethical drug by the Ministry of Health, Labour and Welfare of Japan (MHLWJ). The quality and quantity of the ingredients of kakkonto is standardized by MHLWJ. Kakkonto (Product code; TJ-1, Lot Number 2040001040 and 2120001010) used in the present study was purchased from Tsumura (Tokyo, Japan). Kakkonto is composed of seven dried medicinal herbs: 23.5% *Puerariae Radix*, 18.8% *Cinnamomi Cortex*, 17.6% *Zizyphi Fructus*, 11.8% *Paeoniae Radix*, 17.6% *Ephedrae Herba*, 5.9% *Zingiberis Rhizoma* and 11.8% *Glycyrrhizae Radix*. According to the manufacturer’s information, the seven medical herbs were extracted with hot water, and the extract solution was separated from the insoluble waste and concentrated by removing water under reduced pressure. Spray-drying was used to produce a dried extract powder. The chemical pattern of the extract obtained by three-dimensional HPLC analysis was shown in S1 Fig. The main bioactive compound in kakkonto is puerarin, which is an isoflavonoid derived from Puerariae Radix and possess many pharmacological effects such as antiinflammation, vasodilation, neuroprotection, antioxidant and anticancer [29].

### Experimental protocol of the murine food allergy model

Induction of FA was performed as previously described [21, 22]. Briefly, mice were sensitized twice at a 2-week interval with 100 μg ovalbumin (OVA, albumin from chicken egg white grade V, Sigma-Aldrich, St. Louis, MO, USA) in the presence of 2 mg aluminum hydroxide (Thermo Scientific, Rockford, IL, USA) as an adjuvant by intraperitoneal injection. Two weeks after the systemic priming, the mice were repeatedly given 50 mg OVA (crude ovalbumin powder; Nacalai Tesque, Kyoto, Japan) dissolved in water with intragastric feeding needles three times per week. Diarrhea was assessed by visually monitoring mice for up to 1 h following the intragastric challenge.

### Administration of OIT and combined therapy with kakkonto

**Fig 1:**
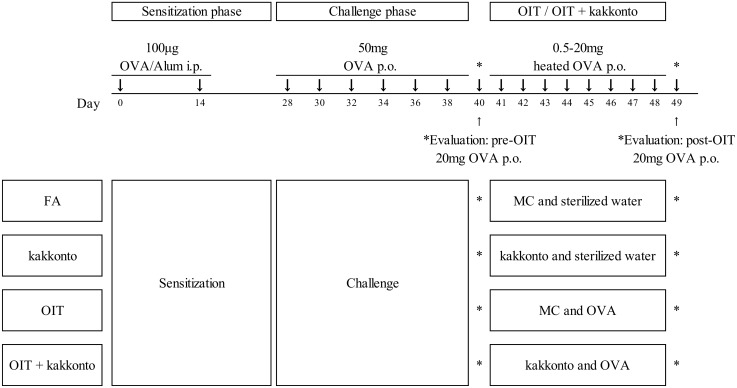
Outline of the experimental design. OVA-sensitized BALB/c mice were challenged 3 times per week by oral administration of non-heated OVA solution. After day 40, mice were treated with kakkonto, OIT, OIT+kakkonto or placebo (MC: 0.5% methylcellulose solution and sterilized water) daily for 8 days. One hour before each oral heated OVA challenge, kakkonto or placebo (MC) was orally administrated. Before and after the OIT, mice received a non-heated OVA challenge to assess allergic symptoms (day 40 and day 49, respectively).

As outlined in Fig 1, an experimental OIT treatment for the FA model mice was performed by oral administration with increasing doses (0.5–20 mg/day) of OVA (grade V; Sigma-Aldrich) from day 41 to day 48. OVA was dissolved in sterilized water and heated in a boiling water bath for 1 min. The heated OVA was administrated at 0.5 mg/day on day 41, 1 mg/day on day 42, 2 mg/day on day 43, 4 mg/day on day 44, 8 mg/day on day 45, 12 mg/day on day 46, 18 mg/day on day 47 and 20 mg/day on day 48. Kakkonto (500 mg/kg) was suspended in 0.5% methylcellulose solution and administrated 1 h before each oral heated OVA challenge for a combination therapy. The mice were challenged with 20 mg OVA (non-heated) for evaluation of the clinical symptoms before/after OIT treatment. The symptoms of allergic diarrhea were assessed 1 h after non-heated OVA challenge, and the severity was classified as follows: normal stool, soft stool, loose stool, mild diarrhea, severe diarrhea, and fluid stool. Mice showing profuse liquid stool (mild diarrhea, severe diarrhea, or fluid stool) were recorded as having diarrhea.

### Quantitative real-time PCR analysis

One hour after the post-OIT non-heated OVA challenge, mice were sacrificed by cervical dislocation, and 2 cm of the proximal colon was excised. The proximal colon was flushed with ice-cold saline and frozen in liquid nitrogen. Total RNA was extracted from the proximal colon by Sepasol Super (Nacalai Tesque) according to the manufacturer’s instructions. Reverse transcription was performed using an Exscript RT reagent kit (Takara Bio, Shiga, Japan) and random primers and was followed by real-time PCR. Real-time PCR amplification of IL-4, IL-13, IL-5, GATA3, CYP26B1 (cytochrome P450, family 26, subfamily b, polypeptide 1), Cellular retinoic acid binding protein 1 (CRABP1), aldehyde dehydrogenase family 1, subfamily A1(ALDH1A1), and glyceraldehyde-3-phosphate dehydrogenase (GAPDH) was performed using SYBR Premix Ex Taq (Takara Bio). The primer sequences of primers are shown in S1 Table. Real-time PCR was performed using a Takara TP800 (Takara Bio). The PCR reaction conditions were 10 sec at 95°C, followed by 40 cycles of 5 sec at 95°C and 20 sec at 63°C. The target mRNA was normalized to GAPDH mRNA as an internal control in each sample. The results were expressed as the ratio relative to the FA group average. The data are from a representative experiment that was repeated three times.

### ELISA

One hour after the post-OIT non-heated OVA challenge, the plasma sample was collected under anesthesia to minimize animal suffering and distress. Mouse mast cell protease 1 (mMCP-1) levels in the plasma were assayed as a marker of mast cell degranulation using an ELISA kit purchased from eBioscience (San Diego, CA, USA). The OVA-specific IgE and IgG1 levels in plasma were measured by an ELISA method as references [30–32]. Briefly, microtiter plates were coated with 10 μg/mL OVA (grade V; Sigma-Aldrich) at 4°C overnight. Plasma samples were incubated for 2 h at 37°C. The binding of OVA-specific IgE was detected using biotinylated rat anti-mouse IgE (1:1,000; YAMASA, Tokyo, Japan) and avidin-HRP (eBioscience). The binding of OVA-specific IgG1 was detected using HRP-conjugated sheep anti-mouse IgG1 (1:2,000; Southern Biotech, Birmingham, AL, USA). Endpoint titers of OVA-specific immunoglobulin antibodies were expressed as the reciprocal log2 of the last dilution that showed a level of >0.1 absorbance over the background levels, which had an absorbance peak at 450 nm.

### Microarray and data analysis

Total RNA was extracted from the proximal colon. mRNA was purified from the mixture of total RNA using an RNeasy Mini Kit (Qiagen, Crawley, UK), and the mRNA from 3 mice from each group was mixed. Microarray analysis was performed, as described previously [21], using the Genechip Mouse Gene 1.0 ST array (Affymetrix, Santa Clara, CA, USA). The Genechip was scanned with a GeneChip Scanner 3000 (Affymetrix), and the gene expression was analyzed using GeneChip Analysis Suite Software (Affymetrix). Transcript levels median-centered and log2-transformed are indicated by color code: blue, low; red, high. The data were analyzed using GeneSpring (Silicon Genetics, Redwood City, CA, USA) and Ingenuity Pathway Analysis (IPA; Ingenuity Systems, Redwood City, CA, USA, http://www.ingenuity.com) to extract significant genes, determine the gene ontology, and identify the canonical pathways associated with the differentially expressed genes. The significance of the association between the microarray data and the canonical pathways was calculated using Fisher’s exact test.

### Immunohistochemistry

Immunohistochemical staining was performed according to the procedure described in previous reports [21, 31–33]. Briefly, one hour after the post-OIT non-heated OVA challenge, the proximal colon was excised, fixed by immersion in 4% paraformaldehyde and then embedded in OCT compound. Cutted sections (30 μm) were exposed to antiserum against mMCP-1, a marker of mouse mucosal mast cells (1:5,000; Moredun Scientific, Scotland, UK) and then incubated with Cy3-conjugated sheep anti-donkey IgG (1:200; Jackson Immunoresearch Laboratories). The immunostained sections were examined using a fluorescence microscope (IX71 system; Olympus, Tokyo, Japan) with a U-MWIG3 filter set (Olympus) and photographed using an Olympus digital camera (DP70; Olympus). The histological staining was quantitatively analyzed using ImageJ software.

### Isolation of lamina propria cells, mesenteric lymph node cells and peripheral blood lymphocytes

One hour after the post-OIT non-heated OVA challenge, the colon was excised and flushed with ice-cold saline. Lamina propria (LP) cells were isolated from the colon as previously described [22]. In brief, the colon was dissected into short segments and stirred at 37°C in RPMI 1640 containing 2% fetal bovine serum (FBS) and 0.5 mM EDTA for 20 min. LP cells were isolated with an enzymatic dissociation procedure using collagenase (Wako, Osaka, Japan). Discontinuous Percoll density-gradient centrifugation was performed to purify the LP cells. Single cell suspensions from the mesenteric lymph nodes (MLNs) were prepared by passing them through a 70-μm mesh filter. Peripheral blood lymphocytes (PBLs) were isolated by density centrifugation with Pancoll (density 1.077 g/mL; PAN Biotech, Aidenbach, Germany).

### Preparation of SpDCs and SpT cells and antigen presentation assay

Splenic dendritic cells (SpDCs) and naive CD4^+^ T cells (SpT cells) were isolated from spleen of wild-type BALB/c mice and RAG-2^-/-^ DO11.10 mice, respectively. Spleens were digested with collagenase (Wako) at 37°C for 15 minutes, and single-cell suspensions were obtained by forcing through a 70-μm cell strainer (BD Biosciences, Franklin Lakes, NJ, USA). Cells were isolated and purified using Mouse CD4 T Lymphocyte Enrichment Set-DM (BD Biosciences) or Mouse Dendritic Cell Enrichment Set-DM (BD Biosciences) according to the manufacturer’s instructions. Carboxyfluorescein diacetate succinimidyl ester (CFSE)-labeled SpT cells (2.0 x 10^5^) and SpDCs (2.0 x 10^4^) were co-cultured in complete RPMI 1640 medium, with 10% FBS (Equitech-Bio, Kerrville, TX, USA), 55 μM 2-mercapto ethanol (GIBCO, Grand Island, NY, USA), 100 unit/ml Penidcillin (Invitrogen, Eggenstein, Germany), 100 μg/ml Streptmysin (Invitrogen), 292 μg/ml Glutamine (Invitrogen), 6.0 ng/ml IL-2 (Peprotech, Rocky Hill, NJ, USA), 1.0 ng/ml Recombinant Human TGF-β1 (Peprotech), 10 ng/ml anti IL-4 (Thermo scientific), 10 ng/ml anti IFNγ (Thermo scientific), 1 μM OVA peptide (Hokkaido system science, Hokkaido, Japan) and retinoic acid (RA) (0.3–100 nM) for 5 days in 96 well flat-bottomed plates. Proliferation of naive T cells was assayed by CFSE dilution. These cells were stained for CD4 and Foxp3 as described in “Flow cytometry analysis”. CFSE dilution assay and analysis of Foxp3^+^ T cell population among gated CD4^+^ T cell were performed by flow cytometry.

### Flow cytometry analysis

To analyze cell surface markers by flow cytometry, LP cells were stained for 30 min at 4°C with PE-conjugated anti-mouse CD4 (clone RM4-5; BD Biosciences). Staining for intracellular Foxp3 was performed according to the manufacturer's instructions using a Foxp3 Staining Buffer Set (eBioscience) and APC-conjugated anti-mouse/rat Foxp3 (clone FJK-16s; eBioscience). Cells were then washed in PBS with 1% bovine serum albumin (BSA) and permeabilized for 1 h in Fixation/Permeabilization working solution (eBioscience). Cells were pre-incubated with a mouse FcR blocking reagent (Milteny Biotec, Auburn, CA, USA) for 5 min before staining to prevent antibody binding to the Fc receptor. Staining with isotype control antibodies was performed in all experiments, and the discrimination of positively stained cells was based on data obtained with isotype control antibodies. Flow cytometry analysis was performed using a FACS Canto II (BD Biosciences).

### Statistical analyses

The values are expressed as the mean ± SE of the respective test or control group (FA group). The statistical significance was evaluated using either a Mann-Whitney test or Bonferroni/Dunn post-hoc tests for multiple comparisons. Probability values (P) of <0.05 were considered as statistically significant.

## Results

### Combined therapy of OIT and kakkonto further ameliorates the allergic symptom

**Fig 2:**
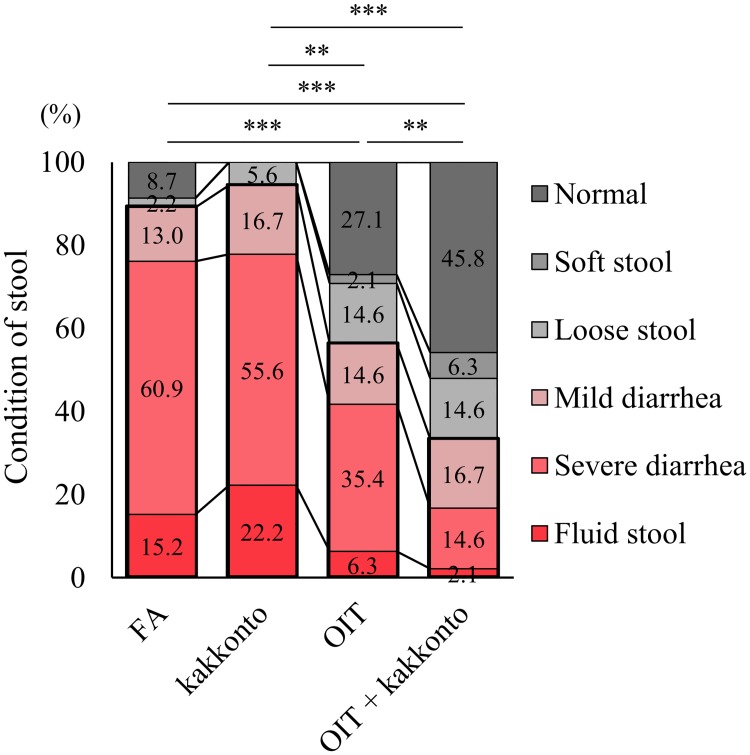
Clinical symptoms in mice under different treatments. OVA-sensitized mice were treated with OIT, OIT and kakkonto combined therapy (OIT+kakkonto), kakkonto alone (kakkonto) or placebo (FA). One hour after the non-heated OVA challenge on day 49, the severity of allergic diarrhea was assessed (kakkonto n = 18, FA n = 46, OIT n = 48, OIT+kakkonto n = 48, **P<0.01, ***P<0.001).

To estimate the therapeutic effects of OIT in mice with already established allergic symptoms and to determine the clinical utility of kakkonto in a combination therapy with OIT, BALB/c mice were sensitized and challenged with non-heated OVA. These mice developed significant allergic diarrhea with increased OVA-specific IgE. OIT treatment was performed with oral administration of increasing doses of heated OVA. Allergic symptoms in response to OIT (e.g., allergic diarrhea) were not observed. OIT significantly suppressed the occurrence and severity of allergic diarrhea in mice with an established FA. Compared with OIT, the combined therapy of OIT and kakkonto (OIT+kakkonto) further ameliorated allergic symptoms, while treatment with kakkonto alone did not inhibit allergic diarrhea (Fig 2). The occurrence of diarrhea on day 49 was 89.1% in untreated FA mice, while it was 56.3% and 33.3% in OIT mice and OIT+kakkonto mice, respectively, with significant differences (P<0.001). These results showed that OIT+kakkonto significantly enhanced the therapeutic effect of OIT on the occurrence and the severity of allergic diarrhea (P<0.01).

### Combined therapy of OIT and kakkonto further downregulates the gene expression of Th2 cell markers

**Fig 3:**
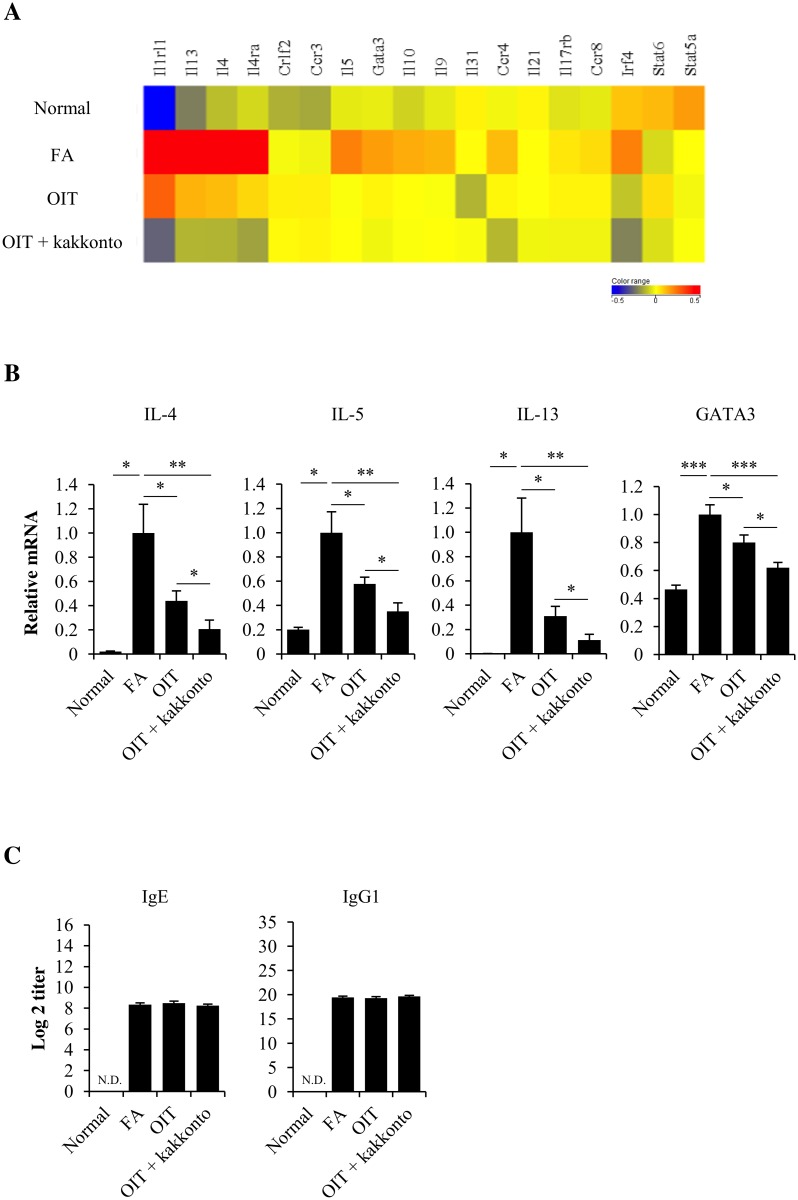
Differential gene expression of Th2 cell markers. (A) Heat map of the expression of genes associated with the Th2 response (subset mentioned in IPA dataset). (B) The IL-4, IL-5, IL-13, and GATA3 mRNA expression levels in the proximal colon. The segment of the proximal colon was obtained 1 h after the non-heated OVA challenge on day 49 (Normal n = 5, FA, OIT and OIT+kakkonto n = 14, *P<0.05, **P<0.01, ***P<0.001). (C) The OVA-specific IgE and IgG1 levels in the plasma was measured using an ELISA 1 h after the non-heated OVA challenge to assess the allergic symptoms on day 49 (Normal n = 5, FA, OIT and OIT+kakkonto n = 20).

To elucidate the mechanism underlying therapeutic effects of OIT and OIT+kakkonto, we examined gene expression in the proximal colon with transcriptome analysis, which indicated a predominant Th2 immune response and mast cell activation in untreated FA mice. However, the selected gene expressions associated with Th2 immune responses (cell surface markers, transcription factors and secreted factors) were decreased in OIT mice, and OIT+kakkonto further downregulated these gene expressions (Fig 3A). Furthermore, the decreased mRNA expression of IL-4, IL-5, IL-13 and GATA3 was verified by real-time RT-PCR (Fig 3B). In contrast, OIT and OIT+kakkonto had no effects on the increased level of OVA-specific plasma IgE and IgG1 in FA mice (Fig 3C).

### Combined therapy of OIT and kakkonto further suppresses the degranulation of mucosal mast cells

**Fig 4:**
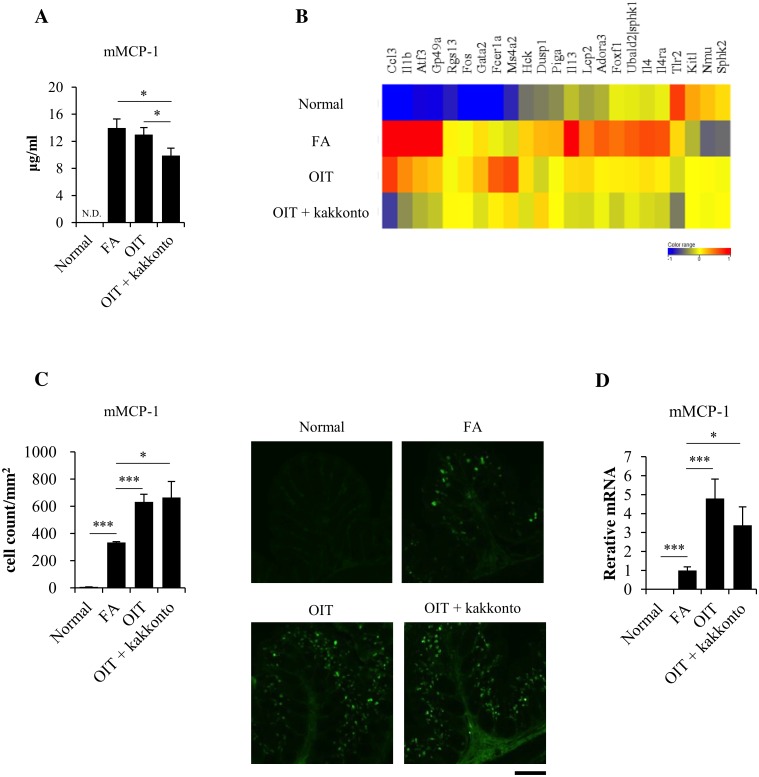
Degranulation and infiltration of mucosal mast cells. (A) mMCP-1 concentration in plasma (Normal n = 5, FA, OIT and OIT+kakkonto n = 20, *P<0.05). (B) Heat map of the expression of genes associated with the mast cell degranulation (subset mentioned in IPA dataset). (C) mMCP-1 positive mucosal mast cells in the proximal colon (n = 5, *P<0.05, ***P<0.001). The scale bar indicates 200μm. (D) mMCP-1 mRNA levels in the proximal colon (Normal n = 5, FA, OIT and OIT+kakkonto n = 14, *P<0.05, ***P<0.001).

The plasma mMCP-1 levels were decreased by OIT and further significantly decreased by OIT+kakkonto (Fig 4A). Furthermore, the transcriptome analysis in the proximal colon showed that mast cell degranulation-related gene expressions were reduced in OIT mice, and OIT+kakkonto further diminished these gene expressions (Fig 4B). We then assessed mucosal mast cell infiltration in the proximal colon. Unexpectedly, the number of mMCP-1 positive cells was significantly increased following OIT and OIT+kakkonto (Fig 4C). This result was supported by the mMCP-1 mRNA expression data in the proximal colon (Fig 4D).

### Combined therapy of OIT and kakkonto further increases Foxp3^+^ CD4^+^ regulatory T cells in the lamina propria

**Fig 5:**
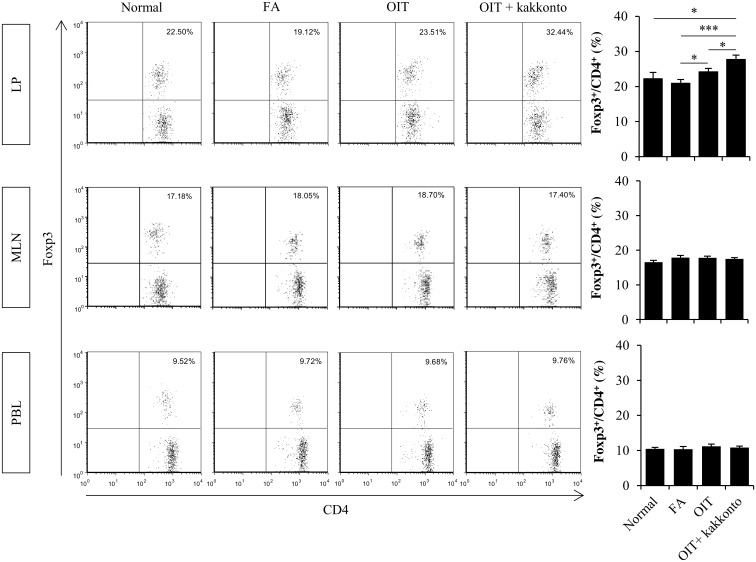
Accelerated induction of Foxp3^+^ CD4^+^ Tregs in the gut by the combined therapy. Flow cytometry analysis of the proportion of Foxp3^+^ CD4^+^ Tregs in the LP (n = 6–20, *P<0.05, ***P<0.001), MLNs (n = 6–9), and PBLs (n = 6–8). Each sample was obtained 1 h after the non-heated OVA challenge on day 49.

Flow cytometry analysis showed that OIT significantly increased the population of Foxp3^+^ CD4^+^ Tregs only in the LP and not in the MLN and PBLs; this population was further markedly and significantly increased by OIT+kakkonto (Fig 5).

**Fig 6:**
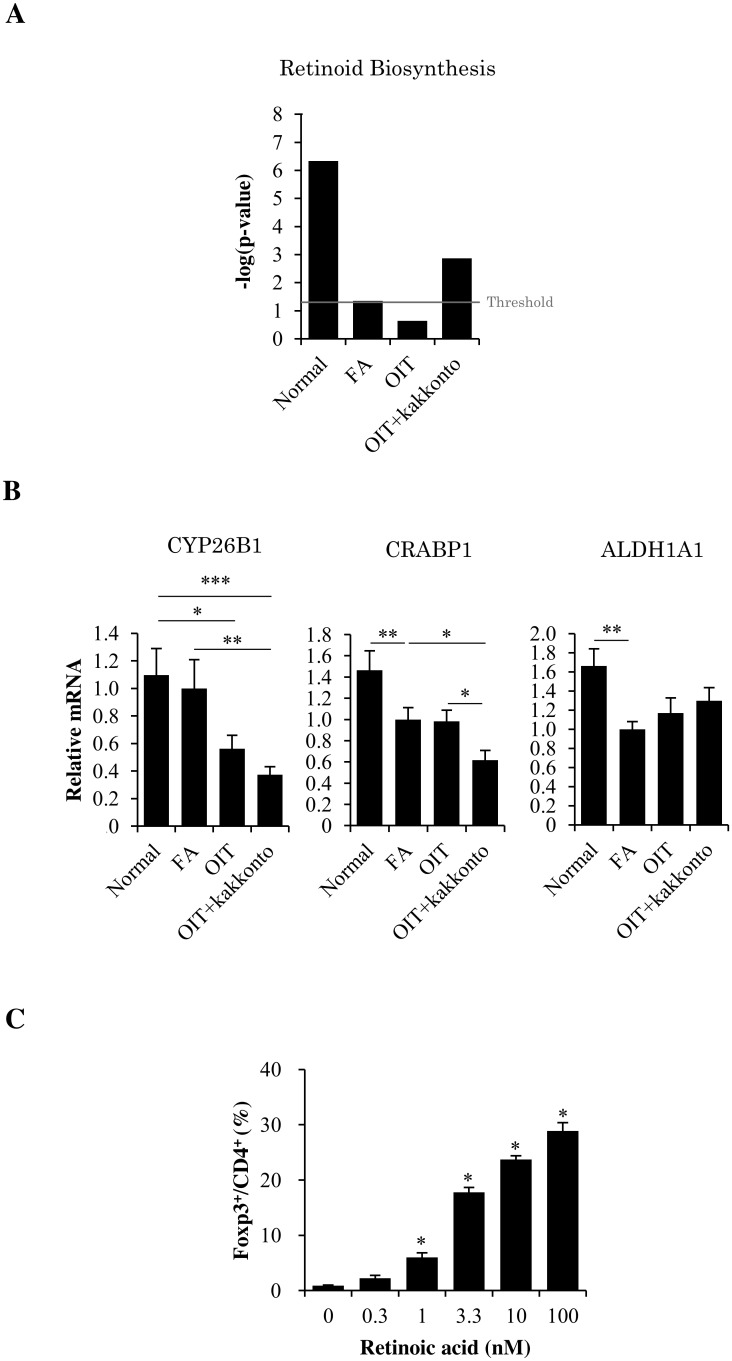
Association of retinoic acid with the combined therapy. (A) Association between our microarray result and the canonical pathway of “retinoid biosynthesis”. Each value is shown as a -log P value, which was calculated using the right-tailed Fisher’s exact test. The threshold line corresponds to the P value of 0.05. (B) CYP26B1, CRABP1 and ALDH1A1 mRNA expression level in the proximal colon. The segment of the proximal colon was obtained 1 h after the non-heated OVA challenge on day 49 (Normal n = 5, FA, OIT and OIT+kakkonto n = 14, *P<0.05, **P<0.01, ***P<0.001). (C) Dose dependency of RA on Foxp3^+^ CD4^+^ Treg differentiation. Naive CD4^+^ T cells from RAG-2^-/-^ DO11.10 mice were co-cultured with SpDCs with the presence of OVA peptide and RA (100 nM, 10 nM, 3.3 nM, 1 nM, 0.3 nM and vehicle) (n = 3, *P<0.05 vs vehicle).

To obtain further insight into the underlying mechanisms of OIT+kakkonto-induced increase of Foxp3^+^ CD4^+^ Tregs in LP, we performed the bioinformatics analysis of the microarray data sets using a pathway and network analysis system IPA. The clusters of biological processes corresponding to genes that were differentially expressed in OIT+kakkonto were shown to be involved to a large extent in the canonical pathway of the biosynthesis and degradation of RA (Fig 6A). RA can enhance the conversion of naive CD4^+^ T cells into Foxp3^+^ Tregs [34–37]. Thus, we focused our analyses primarily upon sets of genes with a previous association with retinoid biosynthesis, and measured the mRNA expression levels of CYP26B1, CRABP1 and ALDH1A1, which regulate RA metabolism, using real-time PCR.

The mRNA expressions of the main RA-degrading enzyme CYP26B1 and the RA-binding protein CRABP1 were significantly downregulated in OIT+kakkonto, whereas no differences were observed in the mRNA expression of ALDH1A1, which is capable of activating RA precursors into the active RA metabolite (Fig 6B). To address the involvement of RA in naive CD4^+^ T cell differentiation, RA was added to co-culture of SpDCs and CFSE-labeled naive SpT cells. As shown in Fig 6C, RA significantly elevated the proportion of Foxp3^+^ CD4^+^ Tregs in a dose-dependent manner.

## Discussion

The effectiveness of OIT for FA has been demonstrated in human patients [8–13]; however, the current therapeutic efficacy of OIT is not satisfactory, and the underlying mechanisms have not been elucidated.

Recently, validity of various antigen administration routes including sublingual route [38], subcutaneous route [39], epicutaneous route [40] and eye anterior chamber route [41–45] for the immune tolerance induction has been shown experimentally and clinically. In addition, there is ample evidence indicating that peripheral mucosal immune systems interact with each other through the common mucosal immune system [46]. Therefore, it is suggested that some administration routes for immune tolerance induction are effective in the treatment of food allergy. In fact, it has been reported that OIT and epicutaneous immunotherapy (EPIT) have a promise for the treatment of food allergy [47]. However, to date, OIT is more effective in inducing tolerance than EPIT and the efficacy of EPIT is not enough for the treatment of food allergy [48]. In addition, OIT utilizes the pathway of oral tolerance that is physiological immune responses to ingested food antigens. Therefore, we selected OIT for further studies.

In the present study, the combination of OIT and kakkonto resulted in a much better inhibitory effect on OVA-specific allergic immune responses in the mouse intestine compared to the effects from either OIT alone or kakkonto alone. Hence, we demonstrated that OIT is effective for treating allergic diarrhea in FA mice, and kakkonto markedly enhances the therapeutic effect of OIT.

Previous studies revealed that intestinal mucosal immunity participates in the development of FA [49–53]. Our previous results indicate that allergic symptoms result from the enhancement of Th2 intestinal immune responses in our FA model [21, 22]. In addition, OIT mice significantly displayed down-regulation of Th2 immune response-related gene expressions compared with FA mice, and OIT+kakkonto further downregulated these gene expressions. Taken all together, these findings indicated that the suppressive effect of OIT and OIT+kakkonto is attributed to the diminished Th2 immune responses.

Rapid desensitization of mast cells by allergens has been proposed as the mechanism underlying allergen-specific immunotherapy in various experimental models. A number of mechanisms are thought to be involved in rendering mast cells unresponsive to allergens, even in the presence of allergen-specific IgE [54–56]. To further elucidate the involvement of mast cells in mediating the effects of OIT and OIT+kakkonto, we measured plasma levels of mMCP-1as a marker of mast cell degranulation [25] and observed that the plasma mMCP-1levels tended to decrease in OIT mice and further significantly decreased in OIT+kakkonto mice; these changes were closely correlated with the allergic symptoms and Th2 immune response-related gene expression.

In a previous report, we showed that treatment with pentagalloylglulcose, a constituent of kakkonto, significantly inhibited the degranulation of mucosal mast cells in an *in vitro* study and suppressed FA in our *in vivo* murine model, which was attributed to downregulation of the FcεR1 surface expression on mucosal mast cells [57]. These observations and the current data raise the possibility that kakkonto significantly enhanced the therapeutic ability of OIT by suppressing the degranulation of mucosal mast cells in OIT mice.

Increased proportions of Foxp3^+^ CD4^+^ Tregs were found in patients treated with immunotherapy [23, 24]. Additionally, induction of Tregs in murine models has been postulated to be an essential step in allergen-specific immunotherapy [25–28]. In 2012, Maazi et al. demonstrated that the effects of allergen-specific immunotherapy can be partially explained by Tregs because depletion of Tregs only partially abrogated the suppressive effects induced by the therapy [58]. Furthermore, Hadis et al. have demonstrated that oral OVA administration induces the local expansion of antigen-specific Foxp3^+^ CD4^+^ Tregs in the LP, but neither the MLN nor the peripheral lymph nodes using OT-II T cell adoptive transfer mouse model and that the antigen-specific Foxp3^+^ CD4^+^ Tregs in the LP contribute to the induction of immune tolerance [59]. Therefore, in this study, to further elucidate the immunomodulating effects of OIT and OIT+kakkonto, we assessed the proportion of Foxp3^+^ CD4^+^ Tregs in the LP following these treatments. Indeed, in our models, we found an increase in the proportion of Foxp3^+^ CD4^+^ Tregs following OIT, which was similar to other reports employing different models of allergic disease; notably, this increase was markedly enhanced by OIT+kakkonto. A number of recent reports have demonstrated that RA, the active metabolite of vitamin A, can enhance the differentiation of naive CD4^+^ T cells into Tregs both in vitro and in vivo [34–37]. In addition, oral administration of vitamin A [60] and subcutaneous administration of RA [61] result in increased number of Foxp3^+^ CD4^+^ Tregs in LP, indicating that RA is a positive regulatory factor for the generation of Foxp3^+^ CD4^+^ Tregs in the intestine. In this study, we showed RA-dependent differentiation of Foxp3^+^ CD4^+^ Tregs in the co-culture of DCs and naive T cells. The concentrations of RA in cells and tissues are regulated by RA metabolism. Cytochrome P450 CYP26 enzymes are primarily responsible for RA degradation [62, 63]. Therefore, suppression of CYP26 enzymes can increase endogenous RA concentrations [63, 64], which enhance differentiation of Tregs [65]. Furthermore, CRABP1 can bind to RA, and transfer and present RA to CYP26 for the degradation [34]. In this study, OIT+kakkonto significantly downregulated the mRNA expression of CYP26B1 and CRABP1, suggesting that OIT+kakkonto may increase RA concentration and thereby induce Foxp3^+^ CD4^+^ Tregs in the LP of the colon.

Granulocyte-macrophage colony-stimulating factor (GM-CSF) has a major role in some inflammatory and autoimmune reactions [66], and it has been reported that enhanced GM-CSF production is associates to the exacerbation of atopic dermatitis [67] and rhinitis [68]. In contrast, GM-CSF has been shown to suppress experimental autoimmune diseases in mice by inducing natural Tregs [69–73]. This finding raise the possibility that GM-CSF has an additional or synergistic effect with OIT, kakkonto or OIT+kakkonto, but additional studies are needed to clearly define the mechanisms and pathways by which GM-CSF acts as both an anti-inflammatory/regulatory cytokine and an inflammatory cytokine.

Treg responses to allergens are associated with the suppression of the effector cells of allergic symptoms, such as mast cells, basophils, and eosinophils [54]. These cells essentially require T-cell cytokines for their priming, survival and activity, but the Th2 cells that were suppressed by activated Tregs in FA mice treated with OIT or with OIT+kakkonto did not efficiently provide these T-cell cytokines. Additionally, through direct Treg-mast cell contact, Tregs limited the degranulation of mast cells. Conversely, it has been reported that the FcεRI signaling blockade in mast cells induces functional Tregs, promotes tolerance induction and prevents food allergen sensitization in a murine peanut FA model [74]. Furthermore, adoptive transfer studies show that infusion of Foxp3^+^ CD4^+^ Tregs results in decreased serum mMCP-1 levels in a FA model [75], indicating that constitutive Foxp3^+^ CD4^+^ Tregs are sufficient to modulate the efferent phase of allergic reactions mediated by mast cells. Based on these observations, Tregs in mice treated with OIT or with OIT+kakkonto can inactivate mucosal mast cells, which play the most pivotal role in the pathogenesis of FAs [76] through various mechanisms, thereby inducing tolerance to food antigens and suppressing FAs.

Taken together, the results of the current study demonstrate that kakkonto can enforce the therapeutic effect of OIT, thus alleviating the allergic symptoms in the murine FA model, indicating that the combined therapy might be useful as a therapeutic intervention undertaken to enhance Treg-mediated immune responses, suppress Th2-mediated immune responses and finally inactivate mast cell activities. Thus, our findings could have important implications for the therapeutic use of the combined therapy of OIT and kakkonto in the treatment of FAs.

## Supporting Information

S1 Fig 3D-HPLC chromatogram of kakkonto. (TIF)

S1 Table Primers used for real-time PCR. (DOCX)

S2 Table Clinical trials of OIT for the treatment of food allergy. (DOCX)
