# Supplementary figures and images for: Improvement of Therapeutic Efficacy of Oral Immunotherapy in Combination with Regulatory T Cell-Inducer Kakkonto in a Murine Food Allergy Model
Source: PLoS One. 2017 Jan 20;12(1):e0170577. doi: 10.1371/journal.pone.0170577 (PMC5249179; doi:10.1371/journal.pone.0170577)

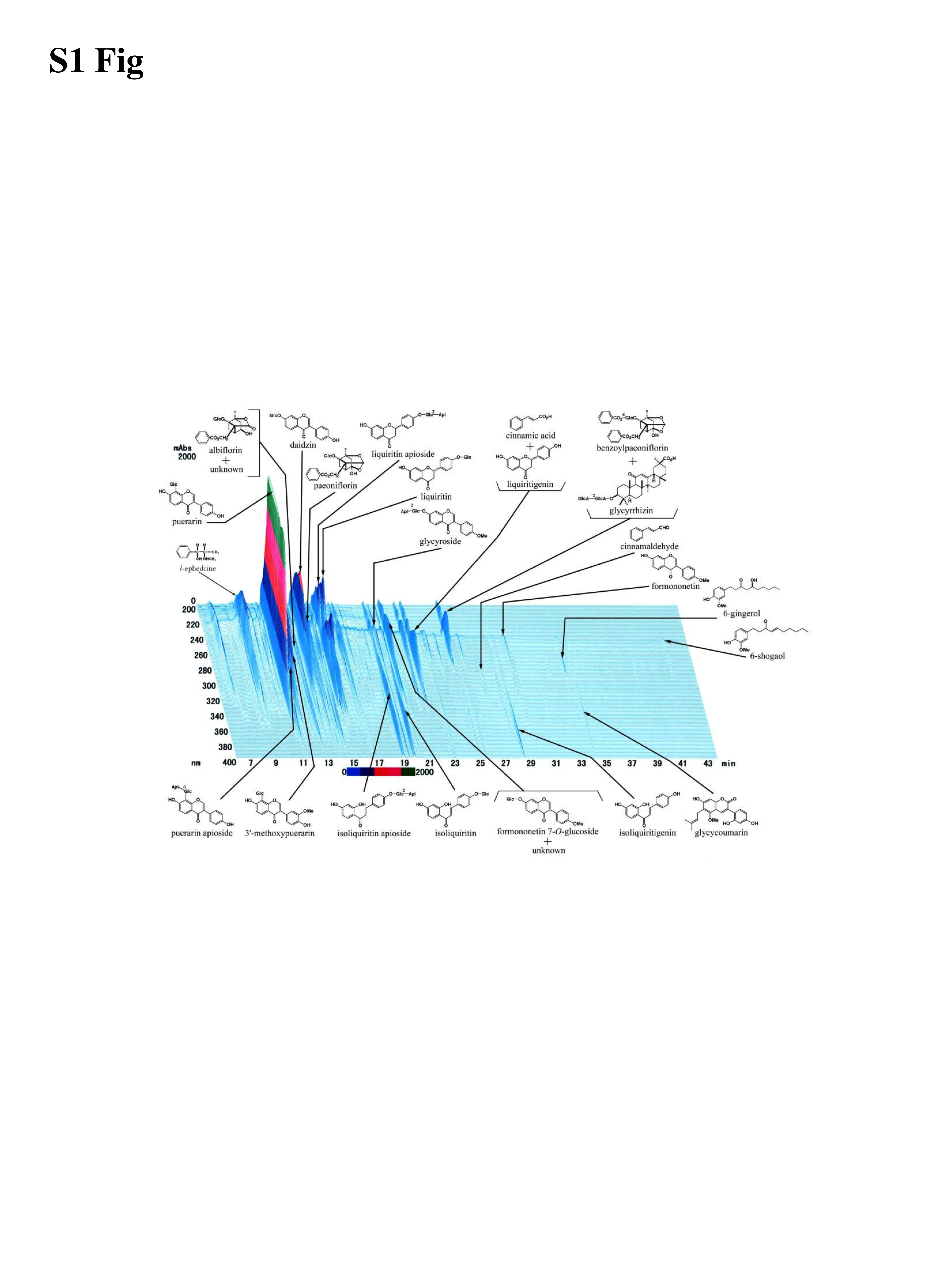

Supplement: S1 Fig — (TIF) [file pone.0170577.s001.tif]
