# Supplementary material for: Improvement of Therapeutic Efficacy of Oral Immunotherapy in Combination with Regulatory T Cell-Inducer Kakkonto in a Murine Food Allergy Model
Source: PLoS One. 2017 Jan 20;12(1):e0170577. doi: 10.1371/journal.pone.0170577 (PMC5249179; doi:10.1371/journal.pone.0170577)
Supplement: S1 Table — (DOCX) [file pone.0170577.s002.docx]

**Supporting Information**

**S1 Table.** **Primers used for real-time PCR**

| Gene | Primer | Sequence |
| --- | --- | --- |
| IL-4 | Forward | 5’-GGTCTCAACCCCCAGCTAGT-3’ |
|  | Reverse | 5’-GCCGATGATCTCTCTCAAGTGAT-3’ |
| IL-5 | Forward | 5’-GAAGTGTGGCGAGGAGAGAC-3 |
|  | Reverse | 5’-GCACAGTTTTGTGGGGTTTT-3 |
| IL-13 | Forward | 5’-GGATATTGCATGGCCTCTGTAAC-3’ |
|  | Reverse | 5’-AACAGTTGCTTTGTGTAGCTGA-3’ |
| GATA3 | Forward | 5’-AGAGATTTCAGATCTGGGCAATGG-3’ |
|  | Reverse | 5’-CAGGGACTGATTCACAGAGCATGTA-3’ |
| mMCP-1 | Forward | 5’-CCATCTGAAGATCATCACGGACA-3’ |
|  | Reverse | 5’-ACATCATGAGCTCCAAGGGTGAC-3’ |
| CYP26B1 | Forward | 5’-CAAGATCCTACTGGGCGAAC-3’ |
|  | Reverse | 5’-GGGCAGGTAGCTCTCAAGTG -3’ |
| CRABP1 | Forward | 5’-GGGGGATGGCCCTAAAACTT-3 |
|  | Reverse | 5’-ACTCGCATCCGGTCATGAAG -3’ |
| ALDH1A1 | Forward | 5’- GGATTCAAGATGTCTGGAAATGGA-3’ |
|  | Reverse | 5’- TGCGACTGTCTTGAGCTCAGTGTA -3’ |
| GAPDH | Forward | 5’-TGACCACAGTCCATGCCATC-3’ |
|  | Reverse | 5’-GACGGACACATTGGGGGTAG-3’ |
