# Supplementary material for: Improvement of Therapeutic Efficacy of Oral Immunotherapy in Combination with Regulatory T Cell-Inducer Kakkonto in a Murine Food Allergy Model
Source: PLoS One. 2017 Jan 20;12(1):e0170577. doi: 10.1371/journal.pone.0170577 (PMC5249179; doi:10.1371/journal.pone.0170577)
Supplement: S2 Table — (DOCX) [file pone.0170577.s003.docx]

**S2 Table. Clinical trials of OIT for the treatment of food allergy**

| Reference | ClinicalTrials. gov Identifier | Official Title |
| --- | --- | --- |
| Longo G et al [8] | NCT01162473 | A Study of the Efficacy of Milk Oral Immunotherapy in Children |
| Meglio P et al [9] | NCT00465569 | A Randomized, Double-Blind, Placebo-Controlled Study of Oral Milk Immunotherapy for Cow's Milk Allergy |
| Buchanan AD et al [10] | NCT00597558 | Treatment of Egg Allergy in Children Through Oral Desensitization (EGG OIT) |
| Jones SM et al [11] | NCT00461097 | Oral Desensitization to Egg With Subsequent Induction of Tolerance for Egg-Allergic Children (CoFAR 3) |
| Clark AT et al [12] | NCT01259804 | Efficacy and Safety of High-dose Peanut Oral Immunotherapy With Factors Predicting Outcome |
| Hofmann AM et al [13] | NCT01074840 | Dendritic Cell Responses to Viral Stimulation in Peanut Allergic Subjects Undergoing Peanut Oral Immunotherapy |
